# Diagnostic accuracy of Raman spectroscopy in oral squamous cell carcinoma

**DOI:** 10.3389/fonc.2022.925032

**Published:** 2022-08-05

**Authors:** Ruiying Han, Nan Lin, Juan Huang, Xuelei Ma

**Affiliations:** ^1^ Department of Biotherapy, West China Hospital and State Key Laboratory of Biotherapy, Sichuan University, Chengdu, China; ^2^ State Key Laboratory of Oral Diseases, National Clinical Research Center for Oral Diseases, Sichuan University, Chengdu, China; ^3^ Department of Hematology, Sichuan Academy of Medical Sciences and Sichuan Provincial People’s Hospital, University of Electronic Science and Technology of China, Chengdu, China

**Keywords:** raman spectroscopy, OSCC, diagnosis, systematic review, artificial intelligence

## Abstract

**Background:**

Raman spectroscopy (RS) has shown great potential in the diagnosis of oral squamous cell carcinoma (OSCC). Although many single-central original studies have been carried out, it is difficult to use RS in real clinical settings based on the current limited evidence. Herein, we conducted this meta-analysis of diagnostic studies to evaluate the overall performance of RS in OSCC diagnosis.

**Methods:**

We systematically searched databases including Medline, Embase, and Web of Science for studies from January 2000 to March 2022. Data of true positives, true negatives, false positives, and false negatives were extracted from the included studies to calculate the pooled sensitivity, specificity, accuracy, positive and negative likelihood ratios (LRs), and diagnostic odds ratio (DOR) with 95% confidence intervals, then we plotted the summary receiver operating characteristic (SROC) curve and the area under the curve (AUC) to evaluate the overall performance of RS. Quality assessments and publication bias were evaluated by Quality Assessment of Diagnostic Accuracy Studies 2 (QUADAS-2) checklist in Review Manager 5.3. The statistical parameters were calculated with StataSE version 12 and MetaDiSc 1.4.

**Results:**

In total, 13 studies were included in our meta-analysis. The pooled diagnostic sensitivity and specificity of RS in OSCC were 0.89 (95% CI, 0.85–0.92) and 0.84 (95% CI, 0.78–0.89). The AUC of SROC curve was 0.93 (95% CI, 0.91–0.95).

**Conclusions:**

RS is a non-invasive diagnostic technology with high specificity and sensitivity for detecting OSCC and has the potential to be applied clinically.

## 1 Introduction

Oral cancer is one of the most common malignant tumors in the head and neck, collectively known as head and neck squamous cell carcinoma (HNSCC) (1). Oral squamous cell carcinoma (OSCC) accounts for 90% of the incidence rate of HNSCC. The mortality rate of HNSCC in the world ranks sixth in the mortality rate of cancer, and the 5-year survival rate after diagnosis is less than 50%, making it a disfigurement disease with poor prognosis (2). The “gold standard” currently in OSCC diagnosis is visual inspection, then an invasive organizational examination or histopathological examination that is invasive and time-consuming (3). Even after successful treatment of primary cancer, there is still a risk of developing recrudescence or second primaries. Thus, screening and detection of early OSCC are the key to reduce the mortality of OSCC patients.

Many non-invasive techniques, such as vital staining techniques, optical imaging devices, and exfoliated cytology tools, have been developed to assist in the screening and early detection of OSCC (4). In 1928, Indian physicist Raman discovered Raman scattering effect, and the spectrum produced by the effect is called Raman spectroscopy (RS). RS can be used to identify the functional groups present in the molecule to provide a specific spectral characteristic of the internal structure and conformation of the cells, referred to as “fingerprint molecules”. Known for its high specificity, high analysis efficiency, and features such as complex samples without dyeing or marking, RS can provide real-time molecular information and high-resolution imaging with a relatively low cost (5). Moreover, biological samples such as tissue, plasma, and saliva can be directly inspected by RS. The non-invasive feature of RS greatly reduces the pain and economic burden of patients and has become a novel tool for cancer diagnosis, treatment, and prognosis evaluation (6). Currently, RS has been proven to have high diagnostic accuracy for multiple types of human cancers, including OSCC (7), breast cancer (8), bladder cancer (9), colorectal cancer (10), and gastric cancer (11).

Although many single-central original studies have been carried out, due to the small number of samples, various diagnostic algorithms and analysis tools, and different RS settings, the previous researches cannot fully reveal the value of RS in OSCC diagnosis. Herein, we conducted this meta-analysis of diagnostic studies to evaluate the overall performance of RS in OSCC diagnosis.

## 2 Methods

### 2.1 Search strategy

A systematic search of the Medline, Embase, and Web of Science databases was performed for English publications for studies from January 2000 to July 2022. Keywords used in database searches were (OSCC) OR (oral cancer) AND (Raman spectroscopy) OR (Raman spectra) OR (Raman spectrum) AND [(histopathology) OR (biopsy)]. Reference lists of retrieved articles and unpublished clinical trials were also examined to identify potential findings.

### 2.2 Study selection

All records were selected for eligibility by 2 reviewers independently, and disagreements were reported to the third reviewer and resolved by discussion. Studies were selected with the following criteria: 1) The samples used should only be human tissue. 2) RS should be an independent diagnostic tool to identify OSCC or differentiate it from normal tissue. 3) Original data including true positives (TPs), true negatives (TNs), false positives (FPs), and false negatives (FNs) should be provided or can be calculated with sensitivity and specificity values. 4) A control group (normal tissues) should be included with a total number of more than 10 samples. 5) Articles should be published in English. Following are the exclusion criteria: 1) Studies used combined diagnostic methods; 2) Studies with animal trial; 3) Irrelevant article types such as reviews and case reports; 3) Studies without providing the exact original data; 4) Studies without a control group; 5) Studies using samples less than 10.

### 2.3 Data extraction and quality assessment

Two reviewers extracted the information from all eligible studies independently according to the selection criteria and organized all of the information into **Table 1**, including principal author, year of publication, country, number of specimens, number of patients, type of RS, diagnostic algorithm, sensitivity, specificity, accuracy, sample type, type of study design (*in vivo* or *in vitro*), spectra, and gold standard. Then, we evaluated the quality of all eligible studies in Review Manager 5.3 software (Cochrane Collaboration, Oxford, England) using Quality Assessment of Diagnostic Accuracy Studies 2 (QUADAS-2) (12).

**Table 1:** Characteristics of the studies included.

Reference	Country	Specimens No.	Patients No.	Type of Raman spectroscopy	Diagnostic algorithm	Sensitivity (%)	Specificity (%)	Accuracy (%)	Sample type	Type of study design	Spectra (nm)	Gold standard
Cals et al., 2015 [19]	Netherland	25	10	SCRA	PCA-KCA	94.6	85.9	90.7	Tissue	*in vitro*	785	Histo
Cals et al., 2016 [20]	Netherland	25	10	SCRA	PCA-LDA	100	66	86	Tissue	*in vitro*	785	Histo
					PCA-hLDA	100	78	91				
Christian et al., 2014 [3]	Germany	72	12	SERDS	PCA-LDA	86.1	94.4	90.3	Tissue	*in vitro*	785	Histo
Connolly et al., 2016 [21]	Ireland	180	36	SERS	PCA-LDA	89	57	73	Saliva	*in vitro*	785	Histo
		120				68	52	60	Oral cells			
Ding et al., 2020 [22]	China	NA	NA	OFRS	DSB-ResNet	97.4	98.8	98.3	Tissue	*in vitro*	785	Histo
Jeng et al., 2019 [23]	China	80	NA	MLRM	PCA-LDA	77.27	86.11	81.25	Tissue	*in vitro*	532	Histo
					PCA-QDA	90.9	83.3	87.5				
Jeng et al., 2020 [24]	China	70	35	MLRM	PCA-LDA	80	85.7	82.9	Tissue	*in vitro*	532	Histo
					PCA-QDA	80	85.7	82.9				
Krishna et al., 2014 [25]	India	603	NA	NIR	MRDF-SMLR	88.3	88.4	88.4	Tissue	*in vivo*	785	Histo
Matthies et al., 2021 [28]	Germany	137	37	SERDS	PCA-LDA	93.7	76.7	88.4	Tissue	*In vitro*	785	Histo
Malik et al., 2017 [26]	India	NA	99	NIR	PCA-LDA	80.9	80	80.2	Tissue	*in vivo*	785	Histo+ follow-up
					LOOCV	78	79.7	79.4				
												
Sahu et al., 2015 [18]	India	328	328	SERS	PCA-LDA	89.7	84.1	87	Serum	*in vitro*	785	Histo
Sharma et al., 2021 [4]	China	131	67	MLRM	PCA-LDA	78.3	100	90.2	Tissue	*in vitro*	532	Histo
Tan et al. 2017 [27]	China	280	280	SERS	PCA-LDA	80.7	84.1	82.5	Serum	*in vitro*	633	Histo
					LOOCV	79.3	82.8	81.1				

SCRA, SpectraCell RA; NIR, near-infrared Raman spectroscopy; SERDS, shifted-excitation Raman difference spectroscopy; SERS, surface-enhanced Raman spectroscopy; MLRM, microscopical laser Raman spectroscopy; OFRS, optical fiber Raman-based spectroscopy; KCA, K-means cluster analysis; LDA, linear discriminate analysis; PCA, principal component analysis; hLDA, hierarchical linear discriminant analysis; LOOCV, leave-one-out cross-validation; DSB-ResNet, diverse spectral band-based deep residual network; QDA, quadratic discriminant analysis; MRDF, maximum representation and discrimination feature; SMLR, sparse multinomial logistic regression.

### 2.4 Statistical analysis

The summary receiver operating characteristic (SROC) curve model of Lee et al. (13) is used for the meta-analysis. TP, FP, TN, FN, sensitivity, specificity, accuracy, and positive and negative likelihood were calculated directly or indirectly using the correct method (14). Then, the pooled diagnostic odds ratio (DOR), publication bias, and the summary receiver operating characteristic (SROC) curve were determined to estimate the area under the curve (AUC) with StataSE version 12 and MetaDiSc 1.4. AUC is correlated with diagnostic value: 0.5 ≤ AUC < 0.7, 0.7 ≤ AUC < 0.9, and AUC ≥ 0.9, respectively representative of a low, moderate, and high diagnostic value (15). The publication bias was calculated with Deeks’ Funnel Plot Asymmetry Test (16). I^2^ statistics was used to evaluate the heterogeneity of the studies included, and the random-effects model would be applied if there was heterogeneity between studies (17). For the studies focused on different kinds of samples such as tissues, serum, oral cells, and saliva, we conducted subgroup analysis to address the heterogeneity of the study effects due to sample type. To avoid the inflation of the type 1 error, the false discovery rate (FDR) method was used to correct *p*-value.

## 3 Results

### 3.1 Study identification

**Figure 1:**
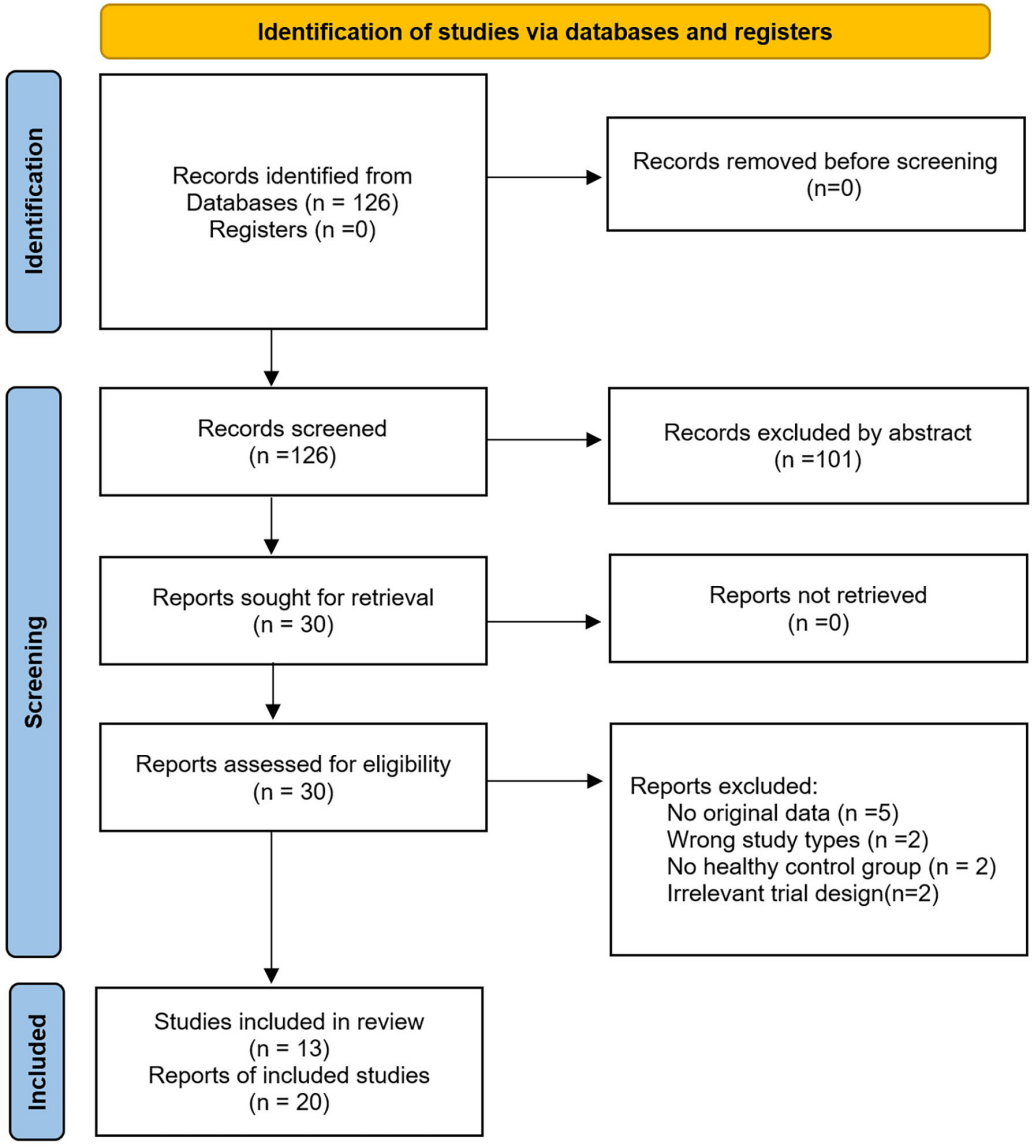
The PRISMA flowchart.

In total, 126 relevant studies were retrieved. After screening for titles and abstracts, we selected 20 studies meeting the criteria for full-text reading. Finally, there were 13 diagnostic studies (3, 4, 18–28) eligible for the pooled analysis. Because several studies included more than one test using different kinds of samples or diagnostic algorithm, there were a total of 20 tests included. **Figure 1** illustrates details of the whole screening process.

### 3.2 Study characteristics, quality assessment, and publication bias

**Table 1** shows the general information of the included studies. All 13 studies were published in English. Most of the studies were published in the recent 5 years, indicating that applying RS for diagnosis is a hot issue recently. Five of the included studies (4, 22–24, 27) were conducted in China, and three (18, 25, 26) were in India. The others were from Netherlands (n = 2) (19, 20), Germany (n = 2) (3, 28), and Ireland (n = 1) (21). Two of the studies (25, 26) are *in vivo*, and the others are *in vitro*. The spectrum was 785 nm in nine studies (3, 18–22, 25, 26, 28), 532 nm in three studies (4, 23, 24), and 633 nm in one study (27). Histopathology was the gold standard of all studies. Principal component analysis (PCA) was the most widely used diagnostic algorithm and was used in every study included. Linear discriminate analysis (LDA) was used in 10 studies (3, 4, 18, 20, 21, 23, 24, 26–28). Other diagnostic algorithms include leave-one-out cross-validation (LOOCV) (18, 26, 27), K-means cluster analysis (KCA) (19), hierarchical linear discriminant analysis (hLDA) (20), diverse spectral band-based deep residual network (DSB-ResNet) (22), quadratic discriminant analysis (QDA) (23, 24), maximum representation and discrimination feature (MRDF) (25), and sparse multinomial logistic regression (SMLR) (25). Different types of RS were used in eligible studies, and studies for each type all account between 1 and 3, including SpectraCell RA (SCRA) (19, 20), near-infrared Raman spectroscopy (NIR) (25, 26), shifted-excitation Raman difference spectroscopy (SERDS) (3, 28), surface-enhanced Raman spectroscopy (SERS) (18, 21, 27), microscopical laser Raman spectroscopy (MLRM) (4, 23, 24), and optical fiber Raman-based spectroscopy (OFRS) (22).

**Figure 2:**
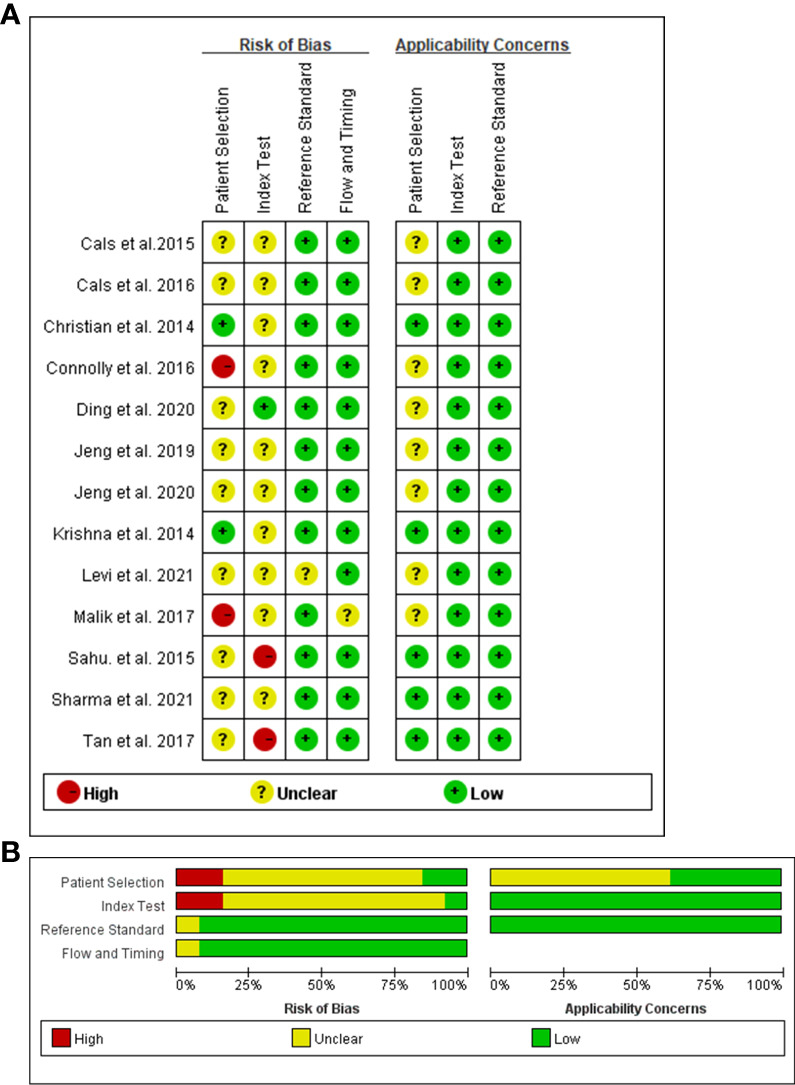
Risks of bias assessment for each included study (n = 12). Risk of bias summary **(A)**. Risk of bias graph **(B)**.

**Figure 3:**
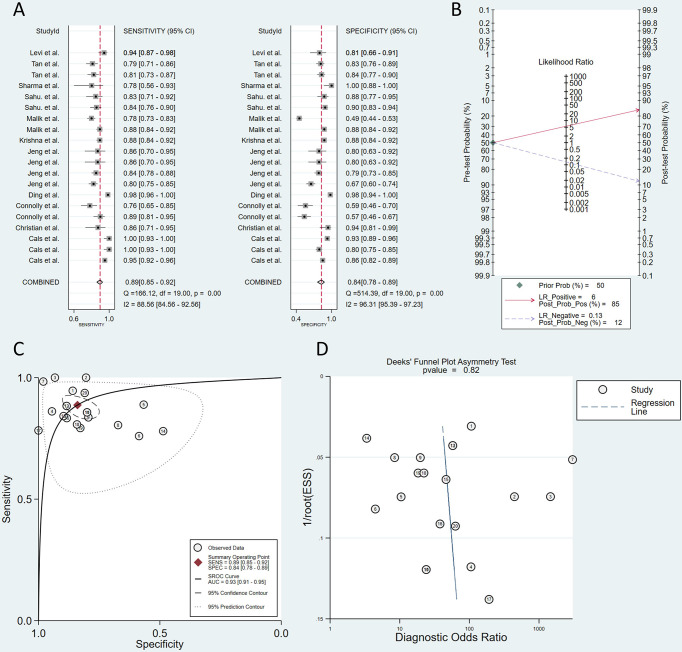
Plots of sensitivity and specificity **(A)**. The positive posterior probability (PPP) and negative posterior probability (NPP) **(B)**. Summary receiver operating characteristic (SROC) curve **(C)**. Deeks’ funnel plot asymmetry test **(D)**.

The QUADAS-2 diagram is shown in **Figure 2**. Most studies conformed to the criteria in QUADAS-2. Some studies’ patient selection and index test items were evaluated as “unclear”, and some literature’s selection of the samples was not random and double-blinded leading to a high or unclear risk of bias. The Deeks’ funnel plot asymmetry test indicated that there was no publication bias (*p* = 0.82; **Figure 3**).

### 3.3 Threshold effect and heterogeneity

We used MetaDiSc 1.4 to analyze the diagnostic threshold effect, and the Spearman correlation coefficient was −0.316 (*p* = 0.187), indicating that there was no threshold effect between the studies.

However, the heterogeneity for sensitivity (Q = 166.12, I^2^ = 88.56) and specificity (Q = 514.39, I^2^ = 96.31) results was significant by using the Q test and I^2^ index. Because the data showed great heterogeneity, multivariate meta-regression was conducted to explore the source of heterogeneity. The countries, diagnostic algorithm, type of study design, type of RS, sample type, and spectra were used as covariates. **Table 2** shows the results of the meta-regression. Type of study design (*in vivo* or *in vitro*) and type of RS (SERS, MLRM, and others) were the sources of heterogeneity.

**Table 2:** Result of meta-regression analysis.

Variable	Coefficient	SD	FDR-*p*-value	RDOR	95% CI
Type of study design	-3.19	0.8169	0.0150	0.04	(0.01;0.25)
Spectra	-0.924	0.5781	0.2768	0.4	(0.11;1.42)
Countries	-0.589	0.4361	0.3054	0.55	(0.21;1.45)
Type of samples	-0.545	0.4773	0.3330	0.58	(0.20;1.66)
Diagnostic algorithm	-0.293	0.5737	0.6196	0.75	(0.21;2.64)
Type of Raman spectroscopy	-1.569	0.472	0.0204	0.21	(0.07;0.59)

S, standard error; RDOR, relative diagnostic odds ratio; CI, confidence interval.

### 3.4 Pooled diagnostic value of raman spectroscopy in oral squamous cell carcinoma diagnosis

A total of 2,051 samples were tested in all of the studies, and 914 patients were included. The sensitivity of the included 12 studies fluctuated between 0.77 and 1. The pooled sensitivity was 0.89 (95% CI, 0.85–0.92), which means that the RS can effectively avoid missed diagnoses. The specificity of the studies ranged from 0.77 to 1 except for two studies using saliva or oral cell as samples. The pooled specificity was 0.84 (95% CI, 0.78–0.89), which means that RS could also avoid misdiagnosis well (**Figure 3**). Pooled sensitivity and specificity determined the SROC curve, and the overall AUC was 0.93 (95% CI, 0.91–0.95), indicating a moderately high overall diagnostic value of RS in OSCC (**Figure 3**). Defining the pretest probability as 0.5, the positive posterior probability (PPP) and negative posterior probability (NPP) were 0.85 and 0.12 (**Figure 3**).

### 3.5 Subgroup analysis

Type of study design (*in vivo* or *in vitro*) and type of RS (SERS, MLRM, and others) tend to be the sources of heterogeneity that may affect the accuracy of the test, so we conducted a subgroup analysis. All of the pooled diagnostic values of subgroup analysis are shown in **Table 3**.

**Table 3:** Pooled diagnostic value of subgroup analysis.

Variable	No.	Pooled sensitivity	Pooled specificity	Pooled PLR	Pooled NLR	Pooled DOR	AUC
*In vivo*	3	0.851	0.697	4.426	0.200	22.178	0.9443
*In vitro*	17	0.884	0.830	5.437	0.158	41.239	0.9336

SERS	6	0.819	0.786	4.009	0.236	17.538	0.8869
MLRM	5	0.824	0.760	3.703	0.238	17.626	0.8963
Others	9	0.908	0.799	8.162	0.084	121.13	0.9645

#### 3.5.1 Type of study design (*in vivo* or *in vitro*)

Three tests (25, 26) were conducted *in vivo*. The pooled sensitivity was 0.851 (95% CI, 0.825–0.874). The pooled specificity was 0.697 (95% CI, 0.667–0.726). The AUC of the SROC curve was 0.9443. The other 17 tests were conducted *in vitro*. The pooled sensitivity was 0.884 (95% CI, 0.870–0.898). The pooled specificity was 0.830 (95% CI, 0.814–0.845). The AUC of the SROC curve was 0.9336.

#### 3.5.2 Type of Raman spectroscopy (surface-enhanced Raman spectroscopy, microscopical laser Raman spectroscopy, and others)

SERS was used in six tests (18, 21, 27). The pooled sensitivity was 0.819 (95% CI, 0.786–0.849). The pooled specificity was 0.786 (95% CI, 0.752–0.817). The AUC of the SROC curve was 0.8869. MLRM was conducted in five tests (4, 23, 24). The pooled sensitivity was 0.824 (95% CI, 0.789–0.855). The pooled specificity was 0.760 (95% CI, 0.718–0.798). The AUC of the SROC curve was 0.8963. Other types of RS were used in nine tests (3, 19, 20, 22, 25, 26, 28). The pooled sensitivity was 0.908 (95% CI, 0.893–0.920). The pooled specificity was 0.799 (95% CI, 0.781–0.816). The AUC of the SROC curve was 0.9645.

In conclusion, *in vitro* detections showed better sensitivity and specificity but lower AUC than *in vivo* ones. The performance of SERS and MLRM was relatively close.

## 4 Discussion

As previous studies revealed, RS has shown its capability in the diagnosis of various types of cancer such as OSCC, lung cancer, and breast cancer. Investigations on the overall performance of RS in the diagnosis of OSCC were of great importance. However, no large-scale study has been carried out to evaluate the value of RS in the diagnosis of OSCC. Therefore, we conducted this meta-analysis to explore the value of RS in the diagnosis of OSCC.

The pooled sensitivity and specificity for RS in diagnosing OSCC are respectively 0.89 and 0.84. Different studies evaluating the same diagnostic indicator can be expressed in the SROC curve. In this meta-analysis, the AUC was 0.93 (95% CI, 0.91–0.95), indicating a moderately high overall diagnostic value of RS in OSCC. Above all, RS has reliable diagnostic performance to differentiate OSCC from normal oral samples according to the pooled sensitivity, specificity, and AUC. When we defined the pretest probability as 0.5, the PPP and NPP were 0.85 and 0.12, and this result demonstrated that RS has the capability to raise the probability of OSCC diagnosis to 85% when positive and lowering the probability of disease to 12% when negative. Therefore, RS is an effective method for the diagnosis of OSCC. Subgroup analysis showed that the pooled sensitivity and specificity of SERS and MLRM group were both over 0.80 and 0.75. Thus, SERS and MLRM both had good diagnostic performance in OSCC. The specificity for the *in vivo* group was slightly lower than the *in vitro* one, which may be explained by the limitation of imaging technology and limited number of studies. However, *in vitro* detection may not be as convenient for clinical operation because of a complicated pretreatment process, and *in vivo* real-time detection of OSCC is a highly potential technology and is worthy of researchers to explore further.

RS has the ability to discriminate normal healthy oral tissue from oral disease states with a fiber-optic probe in the clinical setting, but a major limitation of RS is its dependence on visual detection of morphological or structural lesions. This results in the weakness of Raman signals and even not sensitive enough to merit clinical value. In our study, a technology called “surface-enhanced Raman spectroscopy” showed good diagnostic accuracy. By adsorbing molecules onto nanostructured metal surfaces, SERS can enhance Raman signal by more than 1,000 times, making the possibility of detecting even a single molecule (29). It can also overcome the disadvantage of strong autofluorescence background in common RS. SERS is considered a non-invasive method for OSCC diagnosis using saliva or serum as the samples. In addition, the acquisition and pretreatment process of samples are relatively simple. Blood or saliva test-based screening is more practical and cost-saving for mass screening in nations with a great population like China and India (27). The studies included in this meta-analysis using SERS (18, 21, 27) had a pooled sensitivity and specificity of 0.819 and 0.786, showing little difference with traditional MLRM. Thus, SERS owes great potential in the practical application of RS technology in the clinic and is worthy of further studies and development.

RS not only can be used as a diagnostic tool to differentiate tumors with healthy tissues but also has the potential to distinguish different kinds of oral lesions. Krishna et al. (25) successfully classified the Raman spectra of oral tissue sites into four classes [normal, OSCC, oral submucous fibrosis (OSMF), and oral leukoplakia (OLK)] using the MRDF-SMLR-based diagnostic algorithm. The MRDF-SMLR algorithm showed an accuracy of 85%, 89%, 85%, and 82% in classifying the spectra into the four categories. In the study by Tan et al. (27), using the method of SERS, the PCA-LDA algorithm could classify and diagnose OSCC, mucoepidermoid carcinoma (MEC), and normal groups with a sensitivity and a specificity more than 80%. While the results were encouraging, there are still limitations existing. Firstly, these resulted was not proved by large clinical trials with good design, and still needed to be further studied by subsequent researchers. Secondly, oral disease is various and complicated, and using RS simultaneously identifies the difference between the many kinds of lesions, not just cancer and health, and may result in a greater error rate. Therefore, it still needs to be explored to find how to promote the RS and whether the RS guarantees a high accuracy in the diagnosis of various types and stages of oral lesions.

Automatic diagnosis using RS is inseparable from the application of algorithm. The PCA-LDA is the most common algorithm among them. PCA is a statistical technique that can simplify complicated data by reducing dimensions with the maximum correct rate. However, PCA was not practical for classification cases because of its disability to use any class information extracted from the original data. LDA is a method that can perform a linear transform for feature clusters and change them into forms that can be separated. PCA and LDA are usually combined for dimension reduction and classification of data sets (27). Jeng et al. (23) used two kinds of algorithms, PCA-LDA and PCA-QDA, to test the potential of RS in diagnosing OSCC. The LDA and QDA were used to identify the boundary among different classes. LDA is a good classifier for equal class samples, and QDA performs well in unequal class samples (24). The result revealed that the PCA-QDA model had greater classification efficiency than the PCA-LDA model and could be promoted further. In the study by Cals et al. (20), a two-step PCA-hLDA model was developed. The spectra of adipose tissue and nerve were distinguished from all of the other spectra first, and then the spectra of surface squamous epithelium, CT, muscle, and gland were distinguished from the spectra of OSCC. The PCA-hLDA model showed a better performance than the traditional one-step model. Moreover, RS for recognition and diagnosis based on deep learning is emerging in recent years. Ding et al. (22) created a new classification framework called DSB-ResNet and successfully distinguished tongue squamous cell carcinoma (TSCC) from non-cancerous tissue. Laboratory-level RS can obtain high-quality data, but it may not be suitable for clinical operation because of a complicated pretreatment process. However, deep learning can combine preprocessing, feature extraction, and classification into one architecture to automatically learn the characteristics of data at different levels of abstraction without manual adjustment, greatly simplifying the diagnosis process and achieving higher precision (22). Combined with the DSBResNet framework, a Raman probe device can be used for living samples, and the method proposed in this paper is likely to play a huge potential in the future operation process. However, there are some problems with this study. Firstly, the small amount of data may lead to bias. Secondly, so far, the model had only achieved two classifications, but oral cancer contains many types of cancer, and TSCC is only one of them. Finally, there is still a long way from a potential research on real-time identification to the real clinic. Thus, further detailed biochemical experiments of RS in diagnosis are needed to make the deep learning-based RS technology more practical and smarter.

Although our study suggested that RS showed great potential as a non-invasive, high-accuracy diagnostic tool in diagnosing OSCC, there were still some limitations in the study. Firstly, the gold standard in this research is histopathology, but histopathology can only determine where the sample was taken, thus it is an imperfect gold standard with a corresponding risk of bias. It will be better to combine histopathology with other standards such as follow-up in the study to assess diagnostic accuracy in clinical practice. However, histopathology is still the most effective diagnostic test in clinical practice (30), and it is widely used as the gold standard in our included and other original studies, and only one research included the use of “histopathology + follow up” as its gold standard. So, we still use histopathology as the gold standard in this study, leading to certain limitations. Secondly, diagnosis in the included studies was not made based on predefined criteria or cutoff values, leading to a high risk of overestimation of the diagnostic accuracy by data-driven cutoff selection (31). A characteristic of the Raman spectrum is that the training set is diagnosed by PCA or a similar algorithm to get the difference in the spectrum of normal and cancerous tissues, then the test set can be diagnosed. Therefore, there is no clear diagnostic threshold, and the original study cannot provide the relevant original data. This is an inevitable limitation of the Raman spectrum-related diagnostic meta-analysis and needs further consideration and research in the future. Thirdly, the evidence level of our study might be affected due to a small number of included studies and significant heterogeneity. Although our study included 2,051 samples from 914 patients, the amounts of samples were still insufficient in different groups (such as saliva samples, *in vivo* studies) due to less research in this field, which brought difficulties to the subgroup analysis, even influencing the accuracy of the results. Finally, not all of the studies mentioned that their samples were chosen randomly and double-blindly, resulting in selection bias in the final conclusions. In conclusion, to further verify the role of RS in OSCC diagnosis and promote its clinical practical application, sufficient sample size and randomized and double-blind original studies are required in the future.

## 5 Conclusion

This meta-analysis revealed that RS is a non-invasive diagnostic technology with high specificity and sensitivity for detecting OSCC and has the potential to be applied clinically. Further investigations are also needed to focus on real-time detection using RS with deep learning *in vivo*. Moreover, sufficient sample size and randomized and double-blind original studies are still required in the future to confirm this conclusion.

## Data availability statement

The original contributions presented in the study are included in the article/supplementary material. Further inquiries can be directed to the corresponding authors.

## Author contributions

JH proposed the study concept and was responsible for manuscript editing and manuscript review. RH and NL were responsible for data acquisition, quality control of data and algorithms, data analysis and interpretation, statistical analysis, and manuscript preparation. All authors acknowledge their contributions in this article. All authors contributed to the article and approved the submitted version.

## Conflict of interest

The authors declare that the research was conducted in the absence of any commercial or financial relationships that could be construed as a potential conflict of interest.

## Publisher’s note

All claims expressed in this article are solely those of the authors and do not necessarily represent those of their affiliated organizations, or those of the publisher, the editors and the reviewers. Any product that may be evaluated in this article, or claim that may be made by its manufacturer, is not guaranteed or endorsed by the publisher.
