# Corrigendum: Diagnostic accuracy of Raman spectroscopy in oral squamous cell carcinoma

**DOI:** 10.3389/fonc.2022.1030058

**Published:** 2022-09-21

**Authors:** Ruiying Han, Nan Lin, Juan Huang, Xuelei Ma

**Affiliations:** ^1^ Department of Biotherapy, West China Hospital and State Key Laboratory of Biotherapy, Sichuan University, Chengdu, China; ^2^ State Key Laboratory of Oral Diseases, National Clinical Research Center for Oral Diseases, Sichuan University, Chengdu, China; ^3^ Department of Hematology, Sichuan Academy of Medical Sciences and Sichuan Provincial People’s Hospital, University of Electronic Science and Technology of China, Chengdu, China

**Keywords:** raman spectroscopy, OSCC, diagnosis, systematic review, artificial intelligence

In the published article, there was an error in affiliation 1. Instead of “State Key Laboratory of Oral Diseases, National Clinical Research Center for Oral Diseases, Sichuan University, Chengdu, China”, it should be “Department of Biotherapy, West China Hospital and State Key Laboratory of Biotherapy, Sichuan University, Chengdu, China”.

In the published article, there was an error in affiliation 2. Instead of “Department of Biotherapy, West China Hospital and State Key Laboratory of Biotherapy, Sichuan University, Chengdu, China”, it should be “State Key Laboratory of Oral Diseases, National Clinical Research Center for Oral Diseases, Sichuan University, Chengdu, China”.

In the published article, there was an error regarding the affiliation for Ruiying Han. As well as having affiliation 2, they should also have Department of Biotherapy, West China Hospital and State Key Laboratory of Biotherapy, Sichuan University, Chengdu, China.

In the published article, there was an error. There was a mistake in the time range of the searched literature in Abstract.

A correction has been made to Abstract, *Methods*, 1. This sentence previously stated:

“We systematically searched databases including Medline, Embase, and Web of Science for studies up to March 2022 with no start date limited.”

The corrected sentence appears below:

“We systematically searched databases including Medline, Embase, and Web of Science for studies from January 2000 to March 2022.”

The authors apologize for these errors and state that this does not change the scientific conclusions of the article in any way. The original article has been updated.

## Publisher’s note

All claims expressed in this article are solely those of the authors and do not necessarily represent those of their affiliated organizations, or those of the publisher, the editors and the reviewers. Any product that may be evaluated in this article, or claim that may be made by its manufacturer, is not guaranteed or endorsed by the publisher.

